# PReS-FINAL-2231: A series of 41 mutations of TNFRAF1A

**DOI:** 10.1186/1546-0096-11-S2-P221

**Published:** 2013-12-05

**Authors:** MC Chastang, M Desjonqueres, V Hentgen, P Quartier Dit Maire, G Grateau, I Kone-Paut, I Durieu, J Ninet, P Cochat

**Affiliations:** 1 Hopital Femme Mère Enfant Chu Lyon, Lyon, France; 2 CH Versailles, Versailles, France; 3 Necker, Paris, France; 4 Hopital Tenon, Paris, France; 5 Hopital Kremlin Bicetre, Kremlin Bicetre, Paris, France; 6 Centre Hospitalier Lyon Sud, Lyon, France; 7 HEH, Lyon, France

## Introduction

TRAPS (TNF receptor associated periodic syndrome) is a rare autoinflammatory disease that can touch children and adults. It is caused by the mutation of TNFRSF1A encoding for TNF receptor. The main complication is amyloidosis.

## Objectives

The aim is to increase knowledge about the disease to make the diagnostic easier. Another purpose is to analyse the biotherapy treatment in TRAPS.

## Methods

It consists in a retrospective descriptive multicentre study in French and Belgian hospitals. Data were directly collected thanks to files of patients.

Inclusion criteria are: presence of TNFRAF1A mutation, recurrent symptoms. Exclusion criteria: presence of MEFV mutation.

## Results

We have included 25 kids and 16 adults (isolated cases and 9 families), coming from France (45%), south of Europe (22%), north of Europe (10%), Maghreb (9%), and east of Europe (6%). Two kids have homozygous mutation for MEFV and one heterozygous. 19,5% of the patients have had an appendectomy. 26 patients have recurrent fever in their family, among which 22 have TRAPS.

The disease starts mainly before the age of 5 years (61,1%) but for 13,5%, it begins in adulthood. The average of the time of diagnosis (delay between first symptoms and diagnosis) is 12,9 years.

51% of R92Q heterozygous mutation, 10% of T50M, 7% de L67P, 5% C29S, 5% C43S have been encountered. 2% of the patients have R92Q homozygous, 2% Q82R and R92Q heterozygous.

The seizures occur 9,7 times a year on average (<1 to 48 times a year), last 10,8 days on average (1 to 49 days). A trigger exists in 43.9% of the cases. 78% have rheumatologic symptoms, 70,7% arthralgia (mainly knees, spine, elbows), 22% arthritis (small and big joints). 24,4% have chest pain, 7,3%serositis. Dermatological symptoms (70,7%) are frequent (56,1% rash). Lots of patients have abdominal pain (70,7%), myalgia (65,7%), asthenia (48,8%). Headache is present in 39% of this population. Only 3 patients have periorbital oedema. Between the seizures there is no symptomatology, but in 24% of the cases inflammatory syndrome persists.

We note the interest to dose the Serum Amyloid A to detect the activity of disease between the crises.

The screening of proteinuria was positive in 29% of the cases but no amyloidis is reported.Correlation between R92Q mutation and hematologic symptoms (splenomegaly, adenopathy) was found between genotype and phenotype.

Corticosteroids were used for treatment of seizures. Only 9 patients were treated by biotherapy. Etanercept was efficient in a first time, but not always in the long term. Anakinra always allowed remission.

## Conclusion

77%of this population of patients with TNFRSF1A mutation has 3 symptoms among arthralgia, rash, abdominal pain, myalgia, asthenia and headache. Etanercept is not always efficient and Anakinra is a good alternative for the treatment. The inscription of the patients in autoinflammatory disease registers would allow a better knowledge of TRAPS.

## Disclosure of interest

None declared.

